# Aluminium Involvement in Neurotoxicity

**DOI:** 10.1155/2014/758323

**Published:** 2014-08-27

**Authors:** Alessandro Fulgenzi, Daniele Vietti, Maria Elena Ferrero

**Affiliations:** Dipartimento di Scienze Biomediche per la Salute, Università degli Studi di Milano, Via Luigi Mangiagall, 31, 20133 Milano, Italy

## Abstract

The aetiology of neurodegenerative diseases (ND) seems to involve susceptibility genes and environmental factors. Toxic metals are considered major environmental pollutants. Following our study of a case of multiple sclerosis (MS) improvement due to removal of aluminium (Al) and other toxic metals, we have examined the possible relationship between Al intoxication and ND. We used the slow intravenous treatment with the chelating agent EDTA (calcium disodium ethylene diamine tetraacetic acid) (chelation test) to remove Al and detected it in the urine collected from the patients for 12 hours. Patients affected by MS represented 85.6% of total ND. Al was present in 44.8% of cases comprehensive of ND and healthy patients. Al levels were significantly higher in ND patients than in healthy subjects. We here show that treatment of patients affected by Al burden with ten EDTA chelation therapies (EDTA intravenous administration once a week) was able to significantly reduce Al intoxication.

## 1. Introduction

Exposure of human populations to toxic metals can result in damage to a variety of organ systems.

One of the most commonly toxic metals studied, aluminum (Al), is implicated in many diseases. Al is a highly abundant and ubiquitously distributed as environmental and industrial toxicant and is also contained in many food products, being involved in skeletal, haematological, and neurological diseases [1]. Al toxicity is caused by disruption of homeostasis of metals such as magnesium, calcium, and iron (Fe): in fact, Al mimics these metals in their biological functions and triggers many biochemical alterations [2]. In particular, Al both exerts direct genotoxicity in primary human neural cells [3] and induces neurodegeneration, through an increase in Fe accumulation and oxygen reactive species (ROS) production [4]. Al-induced oxidative damage to DNA has been previously associated with neurodegeneration in different regions of rat brain [5]. In addition, more recently Al^3+^ has been shown to provoke transporter-mediated dopamine neuron degeneration in the nematode* Caenorhabditis elegans* [6].

The removal of toxic metal from human body can represent a useful tool to avoid the beginning or progression of many diseases related to metal intoxication.

The methods useful to determine some metal content in biological samples for monitoring purposes were developed some years ago. Indeed, both toxic and essential metals have been assayed in blood, urine, and hair by atomic absorption spectroscopy [7]. Successively, methods for trace-element analysis in human biological materials have been developed and inductively coupled plasma mass spectrometry (ICP-MS) was considered preferable for screening of multiple elements [8]. However, it seems difficult to show metal excess in blood and urine in conditions different from acute metal intoxication. In fact, blood toxic metal increase reflects only recent exposure to metals [9]. After acute exposure, toxic metals rapidly move from blood to many tissues, where they are sequestered, as in central nervous system (CNS). The only way able to remove accumulated toxic metals from human organs is to bind these metals by means of chelating agents, with the aim of forming complexes able to be excreted in the urine. Toxic metal levels can be examined in the urine samples collected from patients, following “challenge” with a chelating agent (“chelation test”). We have selected, among known chelating agents, calcium disodium ethylenediamine tetraacetic acid (CaNa_2_EDTA or EDTA), which was intravenously administered. The stability constants of aluminium and other metals of biochemical interest with various chelating agents including EDTA have been previously studied [10]. The development of a set of metal complex constants served to correlate chemical and functional properties of the metals and suggested that EDTA was able to mobilize aluminium.

In the past, toxic levels of Al have been associated with neurodegenerative diseases (ND). A possible link between Al and Alzheimer's disease has been highlighted [11]. In 1991, treatment with low dose intramuscular desferrioxamine (DFO), a trivalent chelator that can remove excessive iron and/or aluminium from the body, was reported to slow the progression of Alzheimer's disease [12].

In the present work we have decided to study whether Al was involved in neurotoxicity. Indeed we evaluated the Al body burden in patients affected or not by ND. We studied also the possible reduction of this burden following treatments with the chelating agent EDTA.

## 2. Materials and Methods

### 2.1. Study Design and Patient Recruitment

Out of 471 consecutive subjects who had undergone a medical checkup in an outpatient medical center, only 211 were selected and enrolled for this study due to evidence of their Al burden and compliance in following the protocol, for example, receiving chelation therapy once a week by personal choice. The ND examined in this study were multiple sclerosis (MS), amyotrophic lateral sclerosis (ALS), Parkinson's disease (PD), and Alzheimer's disease (AD). Many MS patients had been previously treated with conventional drugs used in such pathology (e.g., immunosuppressant agents, as mitoxantrone and azathioprine, broad-spectrum immunomodulatory agents, as glatiramer acetate and interferon *β*, and monoclonal antibodies, as rituximab and natalizumab). Some MS patients had never been previously treated with drugs. Patients not affected by known diseases (healthy subject or controls) as well as patients affected by nonneurodegenerative pathologies (not ND, which refers to diseases not classified as ND as fibromyalgia) have also been recruited. Some healthy patients who had been previously exposed to environmental or working toxic metals preferred to examine their possible intoxication by evaluating the presence of such metals in hair samples. Indeed, they were excluded from the present study. All patients provided informed consent to participate in this study. They were between 18 and 75 years old.

### 2.2. Chelation Test and Evaluation of Urine Al

Patients have been subjected to the chelation test to show possible Al intoxication. Indeed, they were invited to collect the urine samples before and after the intravenous treatment with the chelating agent EDTA (ethylenediamine tetraacetic acid, e.g., calcium disodium edetate, 2 g/10 mL diluted in 500 mL physiological saline, Farmax srl, Brescia, Italy). EDTA was intravenously slowly administered (the infusion lasted about 2 hours) to the patients. The time of urine collection following chelation lasted 12 h. Samples recovered from such collection were accurately enveloped in sterile vials and transported to the Laboratory of Toxicology (Doctor's Data Inc., St. Charles, IL, USA), where they have been processed. Samples were acid-digested with certified metal-free acids; digestion took place in a closed-vessel microwave digestion system. For sample dilution ultrapure water was used.

To avoid contamination, only plastic materials were used. All laboratory ware (pipette tips, volumetric flasks, etc.) was immersed for at least 48 h in a 10% (v/v) HNO_3_/ethanol solution and, shortly before use, washed with Milli-Q purified water. To avoid contamination from the air, all steps in the preparation of samples and reagents were carried out on a class 100 clean bench [13, 14].

Testing was performed via inductively coupled plasma mass spectrometry (ICP-MS) utilizing collision/reaction cell methods coupled with ion-molecule chemistry, a new reliable method for interference reduction. The method has been recently used for biomonitoring of 20 trace elements in blood and urine of occupationally exposed workers [15]. Certified urine standards and in-house standards were used for quality control and to validate results. To avoid the potentially great margin of error that can result from fluid intake and sample volume, results were reported in micrograms (*μ*g) per g creatinine. Creatinine was measured by reverse-phase high-performance liquid chromatography and was used to correct the total volume of urinary Al for differences in the glomerular filtration rates of individuals at the time of the spot sample [16]. The research program entitled “Effects of Chelation Therapy with EDTA in Patients Affected by Pathologies Related to Exposition (Acute or Chronic) to Toxic Metals” has been approved by Ethical Commitment of The University of Milan (Italy) (number 64/2014).

### 2.3. Clinical Evaluation of Patient's Symptom Improvement in MS

In the absence of a diagnostic test specific for MS, the neurological community has adopted diagnostic criteria which were replaced in the time [17]. Magnetic resonance imaging (MRI), analysis of cerebrospinal fluid, and visual evoked potential, added to clinical diagnosis, have been considered to present limitations of sensitivity and specificity. Successively MRI has gained an importance. However, the diagnosis of improvement in patient's symptoms is currently based on clinical criteria, as reduction of neurological disability (paresthesia, gait ataxia, spasticity, optic neuritis, and bladder dysfunction) and fatigue. Sometimes, symptoms of ALS, as paresis, muscle atrophy, and dysarthria, are associated with MRI and cerebrospinal fluid abnormalities typical of MS. Indeed, we have considered the improvement of patient's symptoms the recover from clinical disability, for example, ability to work, reduction of spasticity, relapse delay, and/or fatigue disappearance.

### 2.4. Effect of EDTA Chelation Therapy on Al Intoxication

Patients who revealed Al intoxication (by examination of its levels in urine samples) were subjected to EDTA chelation therapy. EDTA (2 g in 500 mL physiological saline) was intravenously infused in each patient in about 2 hours. Treatment was given once a week and lasted ten weeks. At the end of treatments urine Al levels were analysed, as previously described.

### 2.5. Data Analysis

Statistical analysis was performed using Microsoft Excel 2010 and IBM SPSS Statistics 20 (IBM Armonk, New York, USA). A logistic regression analysis was used to examine the relative contributions of several variables to the operation outcomes. *P* < 0.05 was considered significant.

## 3. Results

### 3.1. Patient's Characteristics

**Figure 1:**
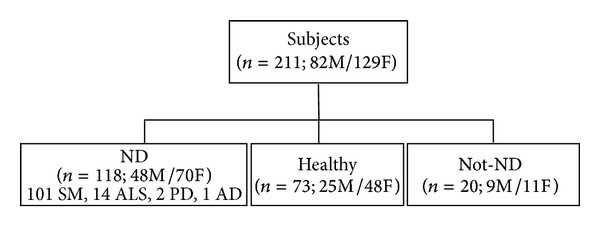
Scheme of enrolled subject's characteristics.

Figure 1 reports the distribution of patients who displayed Al intoxication.

The most represented patients affected by ND were those with MS (85.6% of total ND). Indeed, we compared both the group of MS patients and the group of ND patients with the group of healthy patients.

### 3.2. Al Intoxication

**Figure 2:**
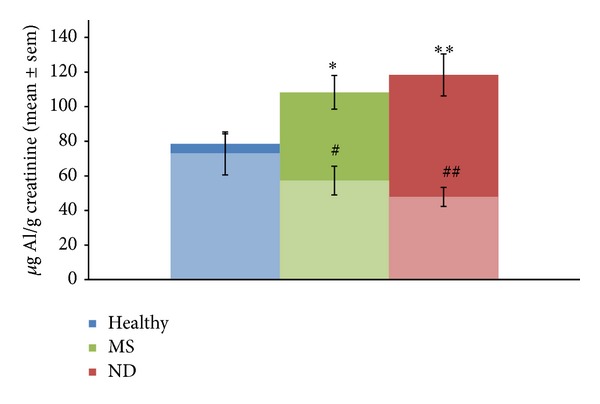
Aluminium (Al) levels evaluated in the urine samples of examinated subjects, following chelation test (dark) and after ten chelation therapies with EDTA (light), expressed as mean ± SEM of *μ*g/g creatinine. The studied subjects were healthy patients, patients affected by multiple sclerosis (MS), and patients affected by all neurodegenerative diseases (ND). The levels of Al in both MS and ND patients were significantly higher with respect to those obtained in healthy subjects following chelation test (*P/**P < 0.05 versus healthy). After chelation therapies with EDTA, the levels of Al were significantly lower than that obtained following chelation test (^#^ *P* < 0.05 versus *P and ^##^ *P* < 0.05 versus **P).

All patients did not show Al intoxication before EDTA challenge (data not shown). All patients affected by ND displayed intoxication by different toxic metals (data not shown). After challenge with EDTA, Al was present in 44.8% of cases comprehensive of ND and healthy patients. The levels of Al intoxication, as obtained from evaluation of *μ*g/g creatinine content of Al in the urine samples collected following the first intravenous treatment with EDTA (chelation test), are reported in Figure 2. The data indicate that Al values were significantly higher in the urine samples of SM and ND patients than in those healthy patients.

### 3.3. Usefulness of EDTA Chelation Therapy

The effect of EDTA chelation therapy is reported also in Figure 2. Indeed, the patients who have shown Al intoxication following chelation test underwent chelation therapy (EDTA intravenous administration once a week). After ten therapies, the levels of Al in the urine samples were further evaluated and compared with that obtained following chelation test. EDTA administration was demonstrated to be significantly efficient in removing Al burden, as shown in Figure 2. Our results showed that reduction in the time of Al intoxication well related with improved clinical conditions of the patients. In fact they presented, at different extent, reduction of neurological disability and fatigue.

Noteworthily, the efficacy of EDTA chelation therapy was more evident in ND than in healthy patients.

## 4. Discussion

Toxic metals, pesticides, and phenols are considered major environmental pollutants [18]. Toxic metals are classified as nonbiodegradable substances, as well as plastics and detergents, because they are not degraded by microorganisms. They represent a global health risk because of their ability to contribute to a variety of diseases. In this context, Al (which is a highly reactive element and ubiquitous environmental contaminant) has been associated with some diseases [1]. In fact, osteomalacia is a skeletal disease related to Al toxic effects, such as phosphate deficiency, Ca-uptake impairment, and dysfunctional osteoblast proliferation [19]. Moreover, Al exposure can impair Fe intestinal absorption, promoting an anemic state [20]. In addition, Al may play an active role in the pathogenetic mechanisms of neurological diseases. In particular, Al has been shown to be responsible for critical neuropathologic lesions in AD and other related disorders for its ability to cross-link hyperphosphorylated proteins [21]. Al has been detected in amyloid fibers in the cores of senile plaques in brains of AD patients [22]. The presence of Al in biological systems could lead to an important prooxidant activity, by promoting superoxide generation through Fenton reaction [23]. More recently, Al removal in AD patients by treatment with DFO has been further proposed [24]. Successful treatment with DFO (both at low and at standard dose) has been performed for Al overload among haemodialysis patients [25]. Moreover, DFO has been shown to be able to exert protective effects in the brain tissue of mice against Al-induced structural and metabolic alterations [26]. However, since some patients can have intolerance to DFO or develop DFO side effects such as allergic reactions, neurological symptoms, or obvious gastrointestinal upset, we decided to use EDTA as a chelating agent. The chelator N-(2-hydroxyethyl) ethylenediamine triacetic acid (HEDTA), similar to EDTA, has been shown to be efficient, also in association with selenium, against Al-induced oxidative stress in rat brain [27].

Elevated urinary excretion of Al and Fe has been previously shown in MS patients [28].

We have previously studied the case of a young man affected by MS, who has been unsuccessfully treated for some years with current therapies [29]. Symptoms revealed by the patient were subacute vision loss, diplopia, and pain with eye movements as the first symptoms of optic neuritis; disturbance of fine motor skills; paresthesia and gait ataxia; bladder dysfunction; and significant tiredness. We examined his levels of toxic metals in the urine, following intravenous “challenge” with EDTA. The patient displayed elevated levels of Al, Pb, and Hg in the urine. Indeed, he was subjected to treatment with EDTA twice a month. Under treatment, the patient revealed in time improved symptoms suggestive of MS remission. In fact, he recovered eye vision and bladder function and paresthesia disappeared as well as tiredness. Because the most represented toxic metal in this patient was Al, we decided to examine the possible relationship of Al intoxication with ND.

Our results show that Al levels measured in urine samples of patients affected by both MS separately studied and total ND studied were significantly higher than that of healthy patients, as reported in Figure 2. Healthy patients displayed about 80 *μ*g/g creatinine, as mean Al levels, even if normal values are 35 *μ*g/g creatinine. These data suggest that Al intoxication is not necessarily related to onset of ND clinical symptoms. Moreover, control patients are possibly able to limit further Al burden through neuroprotective or antioxidant mechanisms which are absent in ND patients. Clinical evaluations of each patient suggested the presence of an important relation between Al intoxication and impairment of movements, paresthesia, ataxia, and other symptoms displayed by subjects affected by ND. Indeed, the patients who displayed maximal values of Al in the urine sample displayed also the most serious features of disease at clinical level. The objection that mobilizing (by chelating agents) Al from relatively safe sites such as bone and depositing this highly neurotoxic metal in the CNS can be dangerous is opposed by the consideration that patients affected by ND were affected by Al burden (responsible for the pathogenesis of the disease) in CNS before chelation. Moreover, the complexes formed by toxic metal with chelating agents are well removed by kidneys. Recent studies demonstrated that severe behavioural motor deficits and loss of the motor neurons through the nervous system resulted when an Al vaccine adjuvant was applied to an animal model. Indeed, mice injected with Al hydroxide showed a significant increase in cell death in the spinal cord and motor cortex, primarily affecting the motor neurons and inducing neuroinflammation. The effects closely resembled the damage seen in human ALS [30].

As recently reported, the immune system also appears to be sensitive to Al exposure [31]. Effects of Al on autoimmunity, oral tolerance, CD4+ and CD8+ expression, hypersensitivity, and erythrocyte immune function are suggestive of its immunotoxicologic activity. It has been suggested that many of the features of Al-induced neurotoxicity may arise in part from autoimmune reactions [30].

Finally, in a recent report by Exley C [32] Al is considered a potential contributor to the onset, progression, and aggressiveness of ND, even if it appears to be difficult to establish when it contributes to disease etiology. However, since Al represents a risk to human health, it is necessary to implement measures to reduce its body burden to the lowest practical limit.

Which strategy for common therapy of injury provoked by toxic metals can be proposed? Intracellular uptake of toxic metals would be adequately prevented by relevant inhibitors (chelators), whereas the ROS generation and ROS-mediated processes would be prevented or ameliorated by relevant antioxidant and scavengers of free radicals and Fe.

In our experience, as shown in previous studies and in the present, removal of toxic metals has induced beneficial effects by improving patient symptoms [29, 33, 34]. No adverse effects were observed from EDTA treatments. Metal removal appeared gradual in the time, and suggested many chelation therapies. In conclusion, in the present study we show that EDTA chelation therapy was able to reduce Al burden in patients affected by ND by ameliorating their clinical conditions. We hope that in the future such treatment will be considered as a useful tool to improve ND patient's symptoms.
